# Clinical Research in Neglected Tropical Diseases: The Challenge of Implementing Good Clinical (Laboratory) Practices

**DOI:** 10.1371/journal.pntd.0004654

**Published:** 2016-11-03

**Authors:** Raffaella Ravinetto, Emilie Alirol, Yodi Mahendradhata, Suman Rijal, Pascal Lutumba, Moussa Sacko, Sayda El-Safi, Kruy Lim, Harry van Loen, Jan Jacobs, Rosanna W. Peeling, Francois Chappuis, Marleen Boelaert

**Affiliations:** 1 Department of Clinical Sciences, Institute of Tropical Medicine Antwerp, Belgium; 2 Clinical Pharmacology and Pharmacotherapy, KU Leuven, Leuven, Belgium; 3 Médecins Sans Frontières United Kingdom, London, United Kingdom; 4 Centre de Recherche Clinique, Hôpitaux Universitaires de Genève, Geneva, Switzerland; 5 Public Health Department, Faculty of Medicine, Gadjah Mada University, Yogyakarta, Indonesia; 6 Department of Internal Medicine, B.P. Koirala Institute of Health Sciences, Dharan, Nepal; 7 Institut National de Recherche Biomédicale, Kinshasa, Democratic Republic of Congo; 8 Department of Tropical Medicine, University of Kinshasa, Democratic Republic of Congo; 9 Institut National de Recherche en Santé Publique, Bamako, Mali; 10 University of Khartoum, Khartoum, Sudan; 11 Sihanouk Hospital Center of Hope, Phnom Penh, Cambodia; 12 Department of Microbiology and Immunology, KU Leuven, Leuven, Belgium; 13 Department of Clinical Research, London School of Hygiene & Tropical Medicine, United Kingdom; 14 Division of Tropical and Humanitarian Medicine, Geneva University Hospitals, Switzerland; 15 Department of Public Health, Institute of Tropical Medicine Antwerp, Belgium; KARI-Trypanosomiasis Res Centre, KENYA

## Introduction

Pharmaceutical research and development (R&D) historically neglected the infectious diseases that mainly or exclusively affect poor communities in low- and middle-income countries (LMICs) [1]. Recently, collaborative clinical research addressing the health needs of LMICs has become more frequent [2], including therapeutic and diagnostic trials for neglected tropical diseases (NTDs), and is often conducted by noncommercial groups. Clinical trials should comply with sound scientific, ethical, and methodological standards, as expressed in a number of international codes [3–5]. The Good Clinical Practices (GCP) codes of the World Health Organization (WHO) and of the International Conference of Harmonization (ICH) provide globally applicable standards for designing, conducting, recording, and reporting clinical trials [4, 5]. Even if primarily meant for the development of new medicines, they are applicable to other clinical investigations with an impact on human safety and well-being [5] and to biomedical research in general [4]. The Good Clinical Laboratory Practices (GCLP) code of the United Nations Development Programme–World Bank–WHO Special Programme for Research and Training in Tropical Diseases (TDR) (2009), adapted from the 2003 GCLP Guidelines of the British Association for Research Quality Assurance (BARQA), provides a GCP-compliant framework for analysis of biological samples [6]. Compliance with GCP provides public assurance that trial participants are protected and data are credible, whereas compliance with GCLP specifically ensures the reliability and integrity of laboratory data.

Compliance with these international codes may seem a daunting task for small, noncommercial research units working in the NTD domain in LMICs, especially when they assume the role of “sponsor,” i.e., the custodian of compliance with legal and ethical frameworks [7]. The challenges of implementing GC(L)P in LMICs have been described by different groups in sub-Saharan Africa, for instance in the fields of a multi-country malaria trial [8] and of vaccine research, respectively [9]. More recently, they have been discussed in the frame of the Ebola outbreak in West Africa [10]. These challenges include, amongst others, (i) contextual constraints (e.g., geographical accessibility, electricity supply, Internet connection, distance from quality suppliers), (ii) the unavailability of research-friendly clinical and laboratory facilities, (iii) the lack of qualified staff (with research and medical experts being reluctant to relocate to remote locations), (iv) the vulnerability of communities, (v) the challenge to ensure post-trial availability and affordability of the research findings, and sometimes (vi) political instability and insecurity. However, these challenges should neither preclude conducting clinical research in NTDs nor lead to lowering the GC(L)P standards.

In this PLOS collection, we share the experience of clinical research on NTD-related syndromes conducted by the NIDIAG consortium (http://www.nidiag.org/) between 2010 and 2015 in Cambodia, the Democratic Republic of Congo, Indonesia, Ivory Coast, Mali, Nepal, and Sudan (ClinTrials.gov identifiers: NCT01589289, NCT01766830, and NCT02105714). This viewpoint article summarizes the main lessons learnt when implementing GC(L)P in NTD clinical research.

### GC(L)P implementation

Because of the features of NTD-endemic areas, implementing GC(L)P requires a significant investment in research capacity. The plans for upgrade of local clinical and laboratory facilities and for staff training should be developed based on thorough pre-study site assessments conducted by clinical, laboratory, and GCP-experts. These visits can often only take place after approval of the research grant and disbursement of the initial budget. In this case, “reasonable flexibility” mechanisms should be negotiated with the funder to allow further adaptation of specific budget lines to local needs [7]. In particular, the local laboratories are often research naïve, so adequate resources should be secured for their upgrade and supervision.

The training plan should carefully consider the knowledge and skills required for different roles. Long-term individual training itineraries, including master and PhD programs, are generally desirable for key staff, e.g., principal investigators and laboratory coordinators from sites in the South. For co-investigators, nurses, laboratory technicians, community workers, etc., ad hoc training modules should be offered on-site. These modules should include comprehensive information on the protocol and overarching research plan to enable every member of the staff (whatever his/her role and hierarchical level) to get an adequate understanding of the importance of their own role and to overcome the sense of disconnect that may be present between medical and nonmedical personnel. The training on protocol, research ethics, and GC(L)P should ideally take place at the trial’s initiation, with the trainer(s) remaining on-site during the first days of recruitment to supervise the team and help them translate procedures into practices [11]. Training should be a continuous process, allowing the maintenance of teams’ capabilities and motivation throughout the trial. This is especially relevant for remote sites, where staff retention is problematic and leads to high turnover. The frequency and intensity of supervision/retraining visits should be tailored to the research complexity and risk, and to the sites’ specific needs.

GCP explicitly require that the quality of the trial be monitored by a qualified person who oversees the trial’s progress to ensure compliance with the protocol, GCP, ethical, and regulatory requirements [4,5]. But, particularly in NTD research, monitoring can have a broader scope, and site visits also provide opportunities for training and mentoring/coaching by external medical and laboratory experts. The latter will play a crucial role in ensuring the quality of data, because a major challenge for research in NTD settings is represented by the upgrade of local laboratories as well as by the harmonization of laboratory quality management systems across sites and countries. The upgrade of local laboratories will include, among others, the improvement of infrastructures; the training of the staff; the setup of an adequate, GCLP-compliant quality management system, including participation to external quality assessment (proficiency testing) [12]; the implementation of measures to mitigate the consequences of extreme climatic conditions [13]; and the setup or improvement of appropriate procedures for biosafety, waste management, etc.

Complementary measures to classical monitoring, such as a risk-adapted approach (http://www.adamon.de/ADAMON_EN/Projectdescription.aspx), may help to keep adequate quality standards, especially when budget constraints limit external visits, but also in other circumstances, e.g., in politically unstable settings where travels may periodically entail security problems. In internal monitoring [14], in particular, a trained member of the team regularly double-checks a subset of data and performs quality checks on consent forms, protocol, and standard operating procedures (SOPs)-compliance. This allows early detection of major or systematic errors. To be valid, internal monitoring should be described in the protocol or SOPs, conducted according to a predefined plan and in consultation with the external monitor, and formally documented. Given the limited psychological independence vis-à-vis the study team, avoiding direct reporting to a more senior member of the study team is suggested. For instance, in the NIDIAG study, the internal monitor (called “quality manager”) reported directly to the sponsor.

Clinical data management capacity is often limited or absent at the local research sites in the domain of NTD. North–South collaborative research provides the opportunity for capacity building in this field. Sufficient resources should be secured not only for hiring and training local data entry staff but also for hiring and coaching local data managers, who may later take in charge the full data management cycle, i.e., from database development to database cleaning and lock.

Trial SOPs should ideally be written in collaboration with the future users. They should be easy to read, practical, and focus on the working instructions and safety. Pretesting in the field is recommended and should involve all the future users, irrespective of their hierarchical level. When possible, job aides should also be developed and pretested to provide a pictorial representation of any trial-specific procedure, such as performance of rapid tests.

### Protecting communities

NTDs mostly affect socially vulnerable populations served by fragile health systems. Social vulnerability [15] has several ethical implications. Wherever access to health care is compromised, the possibility to get access to free care and reimbursements within a trial becomes, in practice, an inducement to participation. Poor households will focus on securing otherwise unavailable health and non-health resources and will underestimate the research-related risks, so becoming vulnerable to exploitation [16].

Protective measures against exploitation should be in place. This requires a solid risk–benefit assessment; the choice—in collaboration with local Ethics Committees—of fair reimbursement schemes and incentives; the early engagement of key local partners, including the use of community meetings to inform and get feedback; and the use of adapted consent tools [17]. Participants should receive adequate medical care, and referral should be facilitated if medical conditions not related to the targeted NTD are diagnosed during the study.

Special attention should be given to local cultural specificities [18]. For instance, in some contexts, the presence of an independent witness at the consent interview is perceived by illiterate patients as a breach of confidentiality rather than a protection mechanism. More broadly, the standard informed consent process is complicated in NTD-endemic contexts by the low literacy rates and by the difficulty of translating research concepts such as randomization into local languages (especially when they do not exist in standardized written forms) [19]. A recent review also confirms that patients in these contexts are less likely to understand the voluntary nature of participation and the freedom to withdraw [20]. New ways to conduct the informed consent process, for instance those relying on the support of audiovisuals and multimedia tools [17], should be investigated on a context-by-context basis to ensure that the consent interview actually empowers research participants rather than being just the fulfillment of a procedural requirement.

Research consortia should also consider what the ethical implications are when trial participants have better access to health care than nonparticipants. To mitigate this, the clinical site must dispose of sufficient staff to ensure adequate care to all patients, either included or not included in the research. But much more should be done by planning broader benefit-sharing measures at the community level. First, trials should result in long-term upgrade of local diagnostic and clinical capacity for the benefit of the health system and populations. Research preparedness and training plans should aim at building capacity, and they should be embedded in a long-term plan for scientific collaboration among North and South research partners that extends beyond the limited timeframe of a specific funding period. Second, to facilitate future access to the interventions developed by research, a comprehensive translation to policy strategy is needed, including prior dialogue with national and international health authorities and an explicit “access” plan (e.g., preferential prices, intellectual property rights measures, etc.). Third, an appropriate framework for sharing data is desirable to allow further analyses and broader scientific collaborations beyond a specific study or a specific consortium, provided that substantial challenges are adequately dealt with, e.g., harmonization of data quality, protection of confidentiality of participants and communities, and fair scientific credit to the researchers and the countries that originated the data [21].

Last but not least, the research protocols should be submitted to an independent Ethics Committee both in the country of the sponsor and in the country/countries where the study is carried out because of their complementary knowledge and competences [15] and to ensure accountability to the public in both contexts.

### Managing research projects

Project management skills are essential in clinical research, but they become vital in remote settings in LMICs. The issue of trial supplies provides a good case to show their importance. Research in NTDs generally involves countries with a poor regulatory environment, so the pre-selection of quality suppliers and the setup of secured supply channels for sensitive items such as concomitant medications and reference diagnostic tests is essential to avoid the risk of poor-quality products [22]. In multicenter/multi-country research, a coordinated and coherent supply plan across sites is needed to avoid bias related to variable quality of medicines, tests, and equipment. The service providers for maintenance of medical and laboratory equipment, including “simple” items like fridges, should be identified upfront, as well as reliable transport agents (possibly with backup mechanisms). Upfront communication with local authorities will ensure a transparent and smooth importation process.

Some contractual negotiations are challenging. For instance, the use and storage of biological samples, especially those exported for analysis and/or biobanking, have been identified as a potential source for liability cases [23]. Long-term storage of biological samples for further research is of paramount importance in NTDs, but it may entail significant cross-border issues, such as benefit sharing and data access. Thus, a fair, equitable, and feasible biobank governance framework will be needed that ensures a fair balance of risks and benefits among all stakeholders [24] and that is translated in adequate contractual arrangements, e.g., the material transfer agreements and the data sharing agreements. To do so, research consortia, and especially the sponsor, should invest in adequate management, legal, and administrative skills just as they do for developing scientific skills [7]. External funding agencies could support ad hoc training on such skills for researchers and managers (both technically and administratively) in the South.

### Final remarks

Building clinical trials capacity at research-naïve sites and institutions in LMICs is always challenging, and this is particularly true for NTD research, which has traditionally received less attention and support compared to other medical fields, such as HIV/AIDS, malaria, and tuberculosis; however, it is feasible [25]. Based on the experience of the NIDIAG consortium, we believe that GC(L)P principles and requirements can and should be implemented in NTD clinical research to ensure protection of patients and communities and to ensure data reliability. This can be achieved, despite the tremendous challenges in NTD-endemic areas, provided that a context-sensitive approach to GC(L)P is adopted, with focus on the needs and priorities of the population and trial sites. In this *PLOS NTDs* collection, we share the set of SOPs developed by the NIDIAG consortium and relate some of the challenges we experienced as case studies.
